# Overall Retention of Methyl Stereochemistry during B_12_-Dependent Radical SAM Methyl Transfer in Fosfomycin Biosynthesis

**DOI:** 10.1021/acs.biochem.1c00113

**Published:** 2021-05-04

**Authors:** Martin I. McLaughlin, Katharina Pallitsch, Gabriele Wallner, Wilfred A. van der Donk, Friedrich Hammerschmidt

**Affiliations:** † Department of Chemistry and Carl R. Woese Institute for Genomic Biology, University of Illinois at Urbana-Champaign, Urbana, Illinois 61801, United States; ‡ Institute of Organic Chemistry, University of Vienna, Vienna 1090, Austria; § Howard Hughes Medical Institute, University of Illinois at Urbana-Champaign, Urbana, Illinois 61801, United States; ∥ Institute of Inorganic Chemistry, University of Vienna, Vienna 1090, Austria

## Abstract

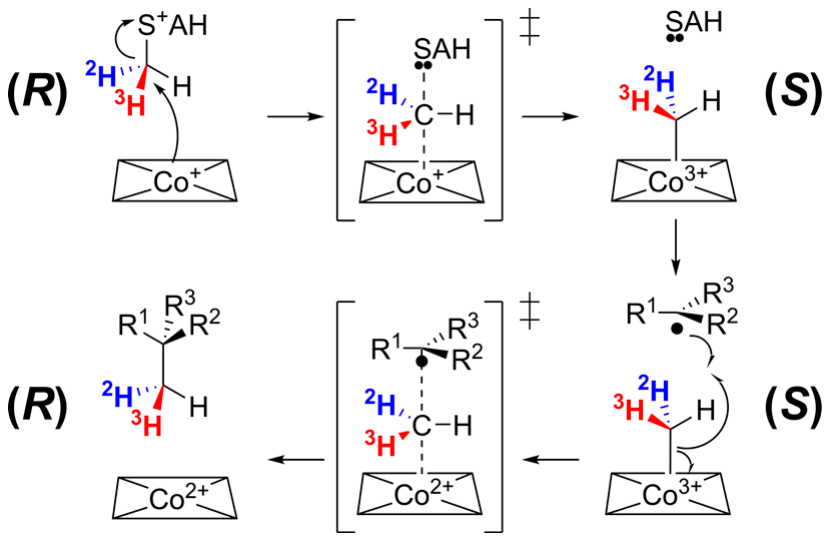

Methylcobalamin-dependent radical *S*-adenosylmethionine (SAM) enzymes methylate non-nucleophilic atoms in a range of substrates. The mechanism of the methyl transfer from cobalt to the receiving atom is still mostly unresolved. Here we determine the stereochemical course of this process at the methyl group during the biosynthesis of the clinically used antibiotic fosfomycin. *In vitro* reaction of the methyltransferase Fom3 using SAM labeled with ^1^H, ^2^H, and ^3^H in a stereochemically defined manner, followed by chemoenzymatic conversion of the Fom3 product to acetate and subsequent stereochemical analysis, shows that the overall reaction occurs with retention of configuration. This outcome is consistent with a double-inversion process, first in the S_N_2 reaction of cob(I)alamin with SAM to form methylcobalamin and again in a radical transfer of the methyl group from methylcobalamin to the substrate. The methods developed during this study allow high-yield *in situ* generation of labeled SAM and recombinant expression and purification of the malate synthase needed for chiral methyl analysis. These methods facilitate the broader use of *in vitro* chiral methyl analysis techniques to investigate the mechanisms of other novel enzymes.

Fosfomycin is a clinically prescribed broad-spectrum and anti-Gram-negative antibiotic produced by two diverging biosynthetic pathways in *Streptomyces* and *Pseudomonas* spp.^1,2^ Its initial discovery^3^ has led to more than 50 years of research into its biosynthesis, mechanism of action, and modes of resistance.^4−13^ In *Streptomyces*, fosfomycin biosynthesis proceeds via the transformation of phosphoenolpyruvate to (5′-cytidylyl)-2-hydroxyethylphosphonate (2-HEP-CMP) catalyzed by Fom1, Fom2, and FomC, three of the six required biosynthetic enzymes (Figure 1).^1,14,15^ Next, Fom3 stereospecifically methylates the sp^3^-hybridized C2 position of 2-HEP-CMP to yield (2*S*)-(5′-cytidylyl)-2-hydroxypropylphosphonate [(2*S*)-2-HPP-CMP].^16−18^ Subsequent hydrolysis of the phosphoanhydride catalyzed by FomD^19^ yields (*S*)-2-hydroxypropylphosphonate [(*S*)-2-HPP], which is oxidized by the non-heme iron epoxidase Fom4^20,21^ to form the final natural product.

**Figure 1:**
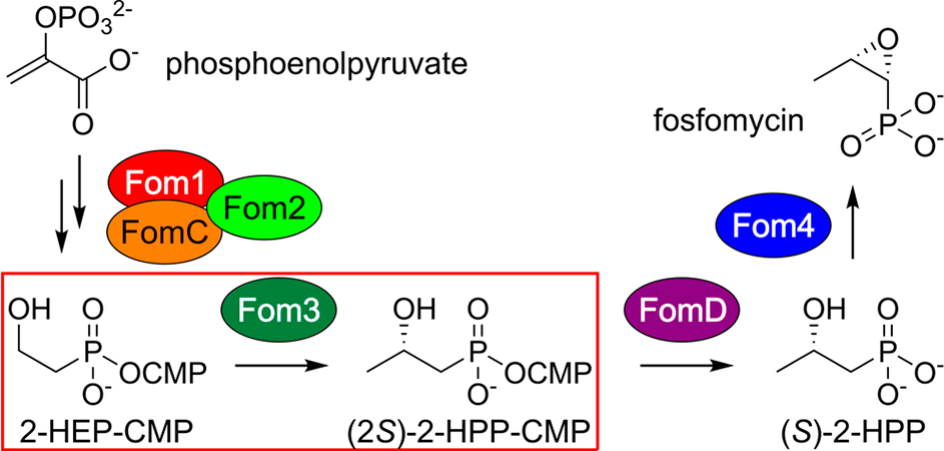
Biosynthesis of fosfomycin in *Streptomyces* spp. CMP = 5′-cytidylyl.

Fom3 (UniProtKB Q56184) is a member of the class B radical *S*-adenosyl-l-methionine (rSAM) methyltransferase family.^22^ These enzymes contain both a cobalamin (B_12_)-binding domain and a domain characteristic of the radical SAM superfamily, which uses a [4Fe-4S] cluster and *S*-adenosyl-l-methionine (SAM) to initiate a diverse range of different chemical transformations.^23^ Class B rSAM methyltransferases attach methyl groups to unactivated carbon centers during the biosynthesis of a variety of molecules, such as gentamicin,^24^ thienamycin,^25^ cystobactamids,^26^ polytheonamides,^27,28^ norcoronamic acid,^29^ and methyl-coenzyme M reductase.^30,31^ Although *in vitro* activity has been obtained for several of these enzymes, many others remain uncharacterized and many more predicted sequences have yet to be assigned a function.^32,33^ Investigating the Fom3 reaction may therefore provide insights into many other important biochemical transformations.

The current working hypothesis for the mechanism of the Fom3 reaction (Figure 2), initially proposed in 2007^14^ and further elaborated in subsequent studies, features methylcobalamin (MeCbl), SAM, and the substrate 2-HEP-CMP bound in the active site of the enzyme. Electron transfer from the reduced [4Fe-4S]^+^ cluster induces reductive cleavage of SAM producing methionine and a 5′-deoxyadenosyl radical (5′-dA•), possibly with the intermediacy of an organometallic species.^34^ The 5′-dA• radical is believed to abstract the pro-*R* hydrogen atom from C2 of 2-HEP-CMP,^16−18,35^ yielding 5′-deoxyadenosine (5′-dA) and a carbon-centered substrate radical. Next, the substrate radical is proposed to attack the methyl group of the enzyme-bound methylcobalamin on the backside of the Co–C bond, leading to homolysis of this bond and formation of a new C–C bond; radical chemistry for methyl transfer from methylcobalamin is supported by model studies by Mosimann and Kräutler.^36^ This step yields the product (2*S*)-2-HPP-CMP with inversion of stereochemistry at the C2 position. Release of 5′-dA, methionine, and (2*S*)-2-HPP-CMP leaves cob(II)alamin in the enzyme active site; MeCbl is then regenerated by one-electron reduction of cob(II)alamin followed by standard S_N_2-type methyl transfer from a second molecule of SAM. Thus, this mechanism for Fom3 has similarities to that proposed for a subset of other radical SAM methyltransferases^24,27,31,37−40^ in that it uses SAM for two distinct types of chemistry for methyl transfer: as a methyl donor for heterolytic methyl transfer (from SAM to B_12_) and as the precursor to 5′-dA• for initiation of radical methyl transfer (from MeCbl to the substrate).

**Figure 2:**
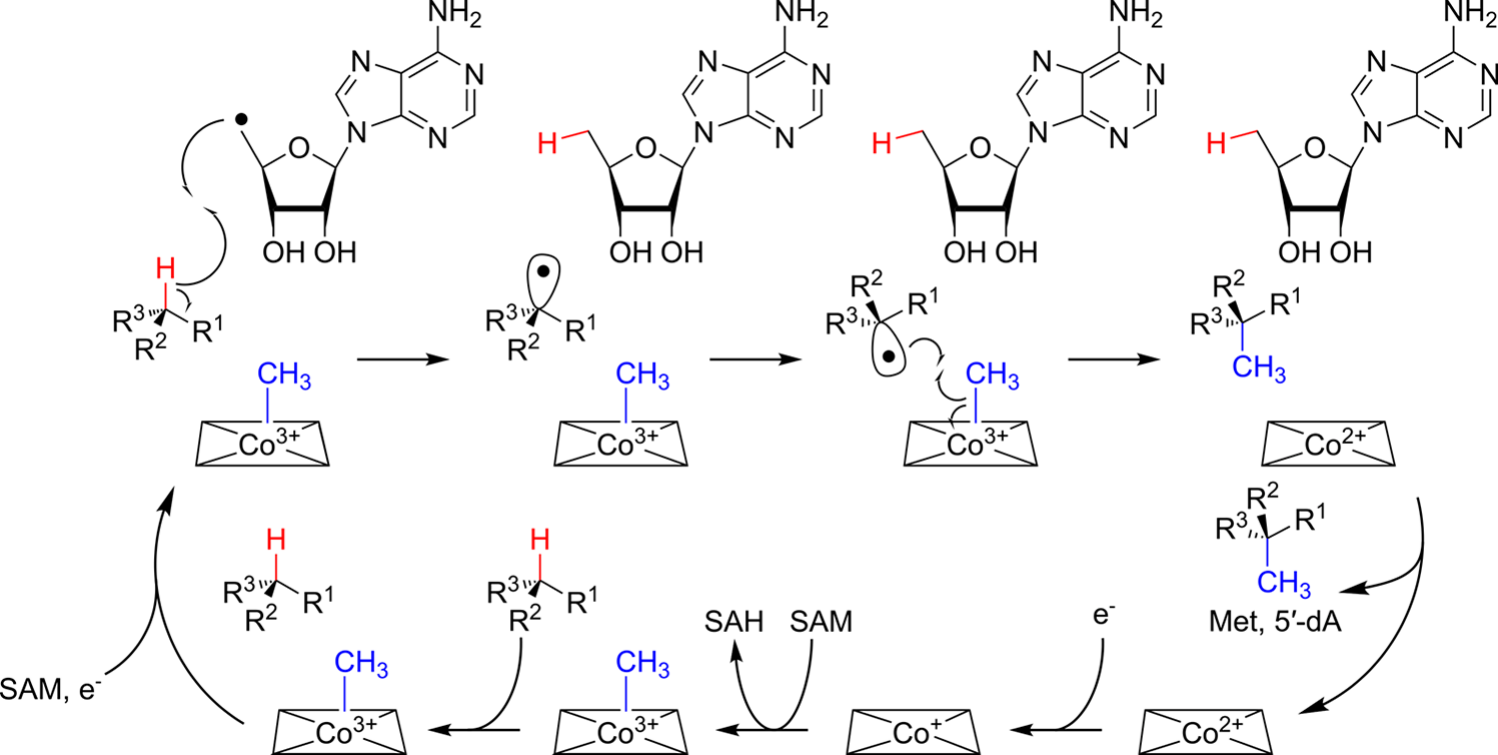
Proposed mechanism for methyl transfer catalyzed by Fom3 and many other B_12_-dependent radical SAM enzymes. The hydrogen atom abstracted from the substrate is colored red; the transferred methyl group is colored blue. For Fom3, R^1^ = H, R^2^ = OH, and R^3^ = CH_2_PO_3_-(CMP). Inversion of configuration is depicted at the methylated carbon atom; in reactions where retention of configuration is observed, hydrogen atom abstraction and methyl transfer are expected to occur on the same face of the substrate.^53^

Previous feeding experiments of fosfomycin-producing organisms with methionine that was labeled with ^1^H, ^2^H, and ^3^H on its methyl group with defined stereochemistry showed retention of configuration between methionine and the final product fosfomycin.^41^ Such chiral methyl groups have been used to investigate the mechanisms of a variety of enzymatic reactions since their first reported characterization by Cornforth and Arigoni in 1969.^42,43^ Investigations using chiral methyl groups have made critical contributions to our understanding of transformations involved in key biological processes ranging from the TCA cycle^44,45^ to steroid biosynthesis,^46,47^ natural product biosynthesis, and methanogenesis.^48,49^ Some of the most striking results have come from feeding microorganisms with chiral methyl-labeled molecules to examine a pathway without knowing the specific target enzymes or reactions *a priori*, such as Arigoni’s demonstration that B_12_ biosynthesis in *Propionibacterium shermanii* occurs with inversion of configuration at all seven methyl groups appended to the corrin ring.^50^ However, such feeding experiments do not directly report on the methyl group during each step of the biosynthesis, and thus, the fosfomycin feeding studies mentioned above can be used only to infer molecular-level insight into the mechanism of the unusual *C*-methylation reaction that converts 2-HEP-CMP to (2*S*)-2-HPP-CMP. The stereochemical course of net methyl transfer has not been established unequivocally with the purified enzyme for any radical SAM methyltransferase, and it is not known whether the *in vitro* conditions used for these reactions faithfully reproduce the *in vivo* process as sometimes unexpected reaction products have been reported.^51,52^ Here we present *in vitro* studies that demonstrate retention of configuration between SAM and 2-HPP-CMP during the Fom3-catalyzed reaction, thereby providing key support for the mechanism presented in Figure 2 and suggesting inversion of configuration during both methyl transfer events in the reaction cycle. Furthermore, the data suggest that the *in vitro* conditions used here for these reactions, which are often harsher than cellular conditions and lead to relatively poor efficiency, do provide a stereochemical outcome that is consistent with studies on cellular biosynthesis.

## Materials and Methods

### Materials

(*methyl*-*S*)- and (*methyl*-*R*)-l-(*methyl*-^2^H_1_)[*methyl*-^3^H_1_]methionine, with ^3^H specific activities of 19.74 × 10^6^ and 20.40 × 10^6^ Bq/mmol, respectively, were synthesized according to published methods^54^ and analyzed for chemical and diastereomeric purity by high-performance liquid chromatography using Chiralpak ZWIX(+) and ZWIX(−) columns (Daicel).^55^ Sodium [2-^14^C]acetate was purchased from Amersham Pharmacia Biotech UK Ltd. (Little Chalfont, U.K.) and dissolved in sterilized water to a concentration of 76 Bq/μL. The Fom3 substrate 2-HEP-CMP was enzymatically synthesized and purified as previously described.^17^ Fumarase (porcine heart, 490 units/mg, 3.2 M ammonium sulfate suspension) and acetate kinase (*Escherichia coli*, 500 units/mL, containing 2 mM ATP) were purchased from Sigma-Aldrich, and phosphotransacetylase (*Bacillus subtilis*, 3000 units/mL, 3.2 M ammonium sulfate solution) was purchased from Megazyme (Bray, Ireland). Acetyl-coenzyme A synthetase (lyophilisate, 5 units) was obtained from r-biopharm (Darmstadt, Germany) as part of the acetic acid food analysis kit and dissolved in 100 μL of deionized water. Sources of other reagents and materials are described in the Supporting Information Materials and Methods.

### Manipulation of Oxygen- and Light-Sensitive Materials

Buffer exchange of SAM synthetase, purification of Fom3, and SAM synthetase and Fom3 reactions were performed in a Coy vinyl anaerobic chamber with an atmosphere of 96–97% N_2_ and 3–4% H_2_ in which the oxygen level was kept below 10 ppm. Fom3 purifications and reactions were performed under red light-emitting diode light (619–628 nm; Sunshine Lighting, New York, NY).

### Nuclear Magnetic Resonance (NMR) and Mass Spectrometry

^1^H and ^13^C NMR spectra were recorded on Bruker Avance III AV 600 and Avance III HD 700 spectrometers in D_2_O (HDO, δ_H_ 4.80) and CDCl_3_ (CHCl_3_, δ_H_ 7.24; CDCl_3_, δ_C_ 77.00) at 25 °C. ^31^P NMR spectra were recorded on an Agilent (Varian) 600 MHz Compact spectrometer in D_2_O at room temperature (approximately 23 °C). High-resolution mass spectra (HRMS-ESI) were recorded using a Bruker Maxis Q-TOF mass spectrometer.

### Scintillation Counting

Sample aliquots were mixed with the scintillation cocktail AquaLight from Hidex (Turku, Finland) to produce a final volume of 20 mL and cooled to ambient temperature for at least 1 h before being counted to prevent chemiluminescence. Scintillation counting was carried out using a Hidex 300 SL liquid scintillation counter equipped with three photomultipliers for use of a TDCR (triple-to-double coincidence ratio) counting method.^56−58^ Typical counting times were between 100 and 1000 s, and 1σ counting uncertainties were <3%. Two aliquots of each sample were counted. The mean values of the counts for ^3^H and ^14^C were used for the calculation of ^3^H/^14^C ratios, and the radiochemical yield (RCY) was determined on the basis of the activity of ^14^C.

### Expression and Purification of Recombinant Enzymes

His_6_-SUMO-Fom3 was expressed in *E. coli* BL21(DE3) containing auxiliary plasmids pDB1282 and btu-pBAD1030C-2 and purified by immobilized metal affinity chromatography (IMAC) according to published methods^17^ with modifications described in the Supporting Information Materials and Methods. His_6_-FomD (UniProtKB O83033) was expressed and purified as described previously.^17^ Codon-optimized synthetic genes encoding the *B. subtilis* SAM synthetase (MetK, UniProtKB P54419) I317V variant and *Saccharomyces cerevisiae* malate synthase (ScMLS1, UniProtKB P30952) inserted between the NdeI and XhoI sites of the pET28a vector (for sequences, see the Supporting Information) were purchased from Twist Biosciences (South San Francisco, CA). His_6_-BsMetK I317V was expressed and purified according to published methods^59^ with modifications described in the Supporting Information Materials and Methods and exchanged into oxygen-free buffer [100 mM HEPES-KOH (pH 8.0) and 10% (v/v) glycerol] before use. His_6_-ScMLS1 was expressed and purified by IMAC, and the N-terminal His_6_ tag was removed with thrombin (details in the Supporting Information Materials and Methods). Concentrations of all purified proteins were estimated by their absorbance at 280 nm using extinction coefficients calculated by ExPASy (web.expasy.org/protparam).

### Sequence of btu-pBAD1030C-2

The authors thank F. Kudo (Tokyo Institute of Technology, Tokyo, Japan) for bringing to our attention the fact that the *E. coli* B_12_ uptake construct btu-pBAD1030C-2 used for our previously published work^17^ may not reflect the published plasmid map (ref (17) and Figure S3). The chloramphenicol resistance gene had duplicated multiple times at some point during plasmid production in *E. coli*, and an additional point mutation in BtuF (P218T) had also occurred; neither mutation had been observed in our initial sequence verification efforts, but we confirmed their presence in our current stock. We corrected the chloramphenicol resistance gene duplication, and the B_12_ content of Fom3 co-expressed with the corrected plasmid was similar to that of Fom3 co-expressed with the uncorrected plasmid. Because this level of B_12_ loading was acceptable for our purposes, we chose not to correct the P218T mutation in BtuF. The new plasmid map for corrected btu-pBAD1030C-2 is shown in Figure S1.

### ScMLS1 Activity Assay

ScMLS1 activity was estimated by monitoring the hydrolysis of acetyl-CoA at 233 nm using a Cary 4000 UV–vis spectrophotometer (Agilent, Santa Clara, CA). Assays contained 100 mM Tris (pH 8.0), 3 mM MgCl_2_, 0.26 mM acetyl-CoA lithium salt (pH 7 with LiOH), 3.6 mM glyoxylic acid (pH 8–9 with NaOH), and 0.05–0.1 unit of enzyme [approximately 50 μL of a 1:300 dilution in malate synthase buffer (see the Supporting Information Materials and Methods)] in a final volume of 1 mL. All assay components except glyoxylate and enzyme were mixed in a Quartz Suprasil cuvette (Hellma Analytics, Müllheim, Germany), and absorbance monitoring was initiated at 233 nm with readings taken every 0.1 s. Glyoxylic acid was added and mixed; the absorbance was then monitored for approximately 10 s to ensure adequate mixing before initiation of the reaction by addition of enzyme. The linear region of the absorbance curve following enzyme addition was fitted, and the slope was converted to concentration units using an estimated Δε_233_ of −4.44 mM^–1^ cm^–1^ for acetyl-CoA hydrolysis. The rate of a control reaction lacking enzyme was subtracted from the reaction rate of the enzyme assays to obtain the corrected enzyme-catalyzed rate. The rates of control reactions lacking glyoxylate were negligible. The activity of the stock solution was calculated in units per milliliter of enzyme stock solution, where 1 unit = 1 μmol/min, using the average of three to five trials.

### Enzymatic Conversion of Methionine to 2-HPP

**Figure 3:**
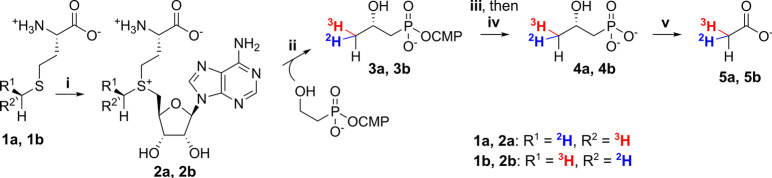
Procedure for conversion of chiral methyl-labeled methionines **1a** and **1b** to chiral acetates **5a** and **5b**, respectively: (i) 10 μM SAM synthetase, 5 mM ATP, 25 mM MgCl_2_, 23 °C, 5 h; (ii) 30 μM Fom3, 250 μM HOCbl, 10 mM DTT, 1 mM methyl viologen, 4 mM NADH, 23 °C, 20 h; (iii) 1 μM FomD, 23 °C, 4 h; enzymes removed via a 10 kDa centrifugal filter; (iv) Dowex 1×8, Cl^–^ form; (v) CrO_3_/H_2_SO_4_, 0 °C for 2 h, then 25 °C for 30 min followed by distillation at ≤1 mbar and neutralization.

Labeled methionines [Figure 3, **1a** and **1b**; 6.9 mg of (*methyl*-*R*)-Met **1b**, 6.6 mg of (*methyl*-*S*)-Met **1a**] were each dissolved in water inside the anaerobic chamber and neutralized with NaOH to create oxygen-free 100 mM stock solutions. A stock solution of l-(*methyl*-^13^C)methionine was created similarly and adjusted to pH 8–9 with NaOH. These solutions were used to assemble 5 mL SAM synthetase reaction mixtures containing 100 mM HEPES (pH 8.0), 50 mM KCl, 25 mM MgCl_2_, 5 mM ATP, 2 mM (10 μmol) methionine, and 10 μM His_6_-BsMetK I317V. After the mixture had been stirred for 5 h at room temperature, some precipitation was visible; Fom3 reaction components were added (250 μM HOCbl, 1 mM 2-HEP-CMP, 10 mM DTT, 4 mM NADH, 1 mM methyl viologen, and 30 μM His_6_-SUMO-Fom3), and the reaction mixtures were stirred for 20 h in the dark. The reaction mixtures were then removed from the anaerobic chamber, and 1 μM His_6_-FomD was added. After reaction of FomD for 4 h at room temperature, enzymes were removed using 10 kDa Amicon centrifugal concentrators (EMD Millipore), and the reaction mixtures were flash-frozen and stored at −20 °C; reaction mixture A (4.853 g, 164274 Bq of ^3^H) was derived from **1a**, and mixture B from **1b** (5.036 g, 168706 Bq of ^3^H). A 200 μL aliquot of the reaction mixture derived from l-(*methyl*-^13^C)methionine was stripped of metals using approximately 100 μL of Chelex 100 resin, sodium form (Sigma), diluted with 100 μL of D_2_O, and analyzed by ^31^P NMR spectroscopy (Figure S2).

### Isolation of (*S*)-2-HPP by Anion Exchange Chromatography

(*S*)-2-HPP obtained from isotopically labeled or unlabeled l-methionine (10 μmol) in enzymatic reaction mixtures was applied to a column filled with Dowex 1×8 resin, Cl^–^ form [1 cm (outside diameter) × 36 cm, 50–100 mesh]. Impurities were removed by washing with deionized water (70 mL), and (*S*)-2-HPP was eluted with 1% (v/v) formic acid (100 mL). The water fraction was discarded for experiments with unlabeled methionine or concentrated under reduced pressure for experiments with labeled methionine and dissolved in an appropriate quantity of water for scintillation counting. The formic acid fraction was lyophilized in both cases, resuspended in water (5 mL), and lyophilized again to remove the remaining traces of acetic acid. Afterward, the lyophilisate was dissolved in distilled water (5 mL) and counted before Kuhn–Roth oxidation. Because the overall Fom3 reaction uses 2 equiv of SAM (Figure 2),^60^ releasing one methyl group as 2-HPP and one as Met, the amount of ^3^H released in the water fraction (Met) and the formic acid fraction (2-HPP) should be equal. The ratio of ^3^H between the water and formic acid fractions after anion exchange chromatography of reaction mixture A was found to be 1:1 (or 1:1.1 in a duplicate experiment), as expected. For unknown reasons, a ratio of 1:1.8 (1:1.3 in a duplicate experiment) was observed for reaction mixture B, but this discrepancy did not affect the stereochemical analysis. Detailed scintillation counting results and radiochemical yields for individual experiments are reported in the Supporting Information Materials and Methods.

### Standard Kuhn–Roth Oxidation: Determination of the Degree of H/D Exchange

(±)-2-Hydroxypropylphosphonic acid × 1.5 cyclohexylamine^61^ (100 mg, 0.346 mmol) was dissolved in deionized water (1 mL) and applied to Dowex 50W×8 resin, H^+^ form [1 cm (outside diameter) × 4 cm, 50–100 mesh]. Elution with water (12 × 1 mL portions) and concentration of the eluate under reduced pressure gave the free phosphonic acid. The residue was dissolved in D_2_O (99.9% D, 1 mL), concentrated again, and again dissolved in D_2_O (5 mL). Chromium trioxide (0.90 g, 9.0 mmol) was added to the stirred solution at ambient temperature and concentrated sulfuric acid (0.57 mL, 10.7 mmol) after cooling to 0 °C. Then, the reaction mixture was stirred at 50 °C (bath temperature) for 3 h and distilled (bath temperature of 160 °C, using a Claisen connecting tube with a plug of glass wool). When the major portion of D_2_O had been distilled off and distillation nearly stopped, H_2_O (5 mL) was added and distillation was continued. Addition of water and distillation were repeated once. Sodium hydroxide (0.1 M) was added to the distillate until it was slightly basic (phenolphthalein). The solution was concentrated under reduced pressure to give sodium acetate (26 mg, 93%) as a nearly colorless salt: ^1^H NMR (700.4 MHz, D_2_O) δ 1.93 (CH_3_), satellite t at 1.917 (*J* = 2.1 Hz) for CH_2_D, and satellite quint at 1.90 (*J* = 2.1 Hz); ^13^C NMR (150.93 MHz) δ 23.30 (CH_3_), 23.06 (t, *J* = 19.6 Hz, CH_2_D), 22.83 (quint, *J* = 19.6 Hz, CHD_2_). The total amount of deuterated acetates was determined by signal heights: 93% CH_3_, 6% CH_2_D, and 1% CHD_2_ by ^1^H NMR spectroscopy and 94%, 5%, and 1% by ^13^C NMR spectroscopy. The thus-obtained sodium acetate was further converted to 4′-(phenyl)phenacyl acetate [68 mg, 78% (see the Supporting Information Materials and Methods)] to verify the obtained results: MS-ESI (accumulation of data for 1 min) 93.5% CH_3_, 5.4% CH_2_D, 1.1% CHD_2_. When the Kuhn–Roth oxidation described above was repeated at 20 °C along with the distillation at a bath temperature of 160 °C, the results were virtually the same.

### Optimized Kuhn–Roth Oxidation to Minimize H/D Exchange

Standard Kuhn–Roth oxidation as described above was modified by performing the reaction at 0 °C (2 h), followed by stirring at room temperature (30 min) and performing the subsequent distillation under reduced pressure (0.5–1.0 mbar, bath temperature of 35–45 °C, 2 × 5 mL of water added for distillation) using the apparatus depicted in Figure S3. The receiver flask was cooled with liquid nitrogen. The distilled acetic acid was adjusted to pH 8–9 with NaOH (for H/D exchange experiments) or KOH (for all other experiments). The solvent was removed by rotary evaporation (for H/D exchange and ^13^C experiments) or lyophilization (for experiments with chirally labeled methyl groups) to yield the solid acetate salt; radioactive samples **5a** and **5b** were then quantified by scintillation counting. When this procedure was performed in D_2_O with unlabeled (±)-2-HPP, the yield of sodium acetate was 25 mg (89%) and the amount of CH_2_D was estimated to be ≤2% by HRMS-ESI of the respective 4′-(phenyl)phenacyl acetate.

### Conversion of Potassium (2-^2^H_1_)[2-^3^H_1_]Acetates **5** to Malates **I**(62)

Potassium acetate **5a** or **5b** (2–3 μmol) was dissolved in 1 mL of carbonate buffer [0.2 M Na_2_CO_3_ (pH 9.3), 8 mM MgCl_2_, and 2 mM K_3_EDTA] and 400 μL of H_2_O. ATP disodium salt (8 μmol, 20 μL of a 400 mM stock), coenzyme A sodium salt (0.6 μmol, 100 μL of a 6 mM stock), sodium glyoxylate (4 μmol, 40 μL of a 100 mM stock), and dl-dithiothreitol (0.6 μmol, 100 μL of a 6 mM stock) were added. The resulting reaction mixture in a glass cylinder [1.5 cm (outside diameter) × 5 cm, with a lid] was spiked with sodium [2-^14^C]acetate (2000–4000 Bq), and the pH was adjusted to 8.7 (0.1 M HCl). Malate synthase (10–30 units), phosphotransacetylase (18–54 units), and acetate kinase (7–21 units) were added. In experiment Ia-3, phosphotransacetylase and acetate kinase were replaced by acetyl-CoA synthetase (5 units) and the Na_2_CO_3_ in the carbonate buffer was replaced by 0.1 M KH_2_PO_4_ (pH 7.4). In all experiments, the reaction proceeded for 2 h at ambient temperature (25–27 °C), with stirring during the first 5 min. Unlabeled malic acid (23 mg) and perchloric acid (8 drops, 70%) were then added to the reaction mixture, and the pH was adjusted to 8–9 with an aqueous KOH solution (0.1 M). The mixture was filtered and loaded onto Dowex 1×8 resin, formate form [1.4 cm (outside diameter) × 11 cm, 100–200 mesh], and successively washed with water (150 mL), 0.2 M formic acid (50 mL), 0.5 M formic acid (50 mL), 0.8 M formic acid (2 × 25 mL fractions), and 1.0 M formic acid (6 × 25 mL fractions). Fractions containing malic acid were identified by TLC on cellulose (75:15:10 Et_2_O:HCO_2_H:H_2_O; *R_f_* = 0.60), combined with the last preceding and first subsequent fractions, concentrated under reduced pressure, and dried (1 mbar). The residue was dissolved in dry acetone (10 mL), concentrated again, dissolved in acetone (1 mL), and filtered through a plug of cotton wool, and the reaction flask was again washed with acetone (2 × 1 mL). The combined acetone filtrates were evaporated in a glass cylinder at ambient pressure and temperature, and the final drying of the residue at 1 mbar furnished usually crystalline malate **I** (20–26 mg), which was dissolved in 1 mL of water. Two 100 μL aliquots were withdrawn for scintillation counting, and the remainder was used for the fumarase reaction. Details for individual experiments are listed in the Supporting Information Materials and Methods.

### Conversion of Malates **I** to Malates **II**

Malate **Ia** or **Ib** (dissolved in 0.8 mL of water) was added to 1 mL of KH_2_PO_4_ buffer (50 mM, pH 7.4). The pH of the resulting solution was adjusted to 7.4 (1 and 0.2 M aqueous KOH, micro pH electrode); fumarase (35 units) was added, and the reaction mixture was stirred for 5 min and then allowed to equilibrate for 3 h at ambient temperature (25–27 °C) before being heated to 90 °C for 3 min. The reaction mixture was then either filtered directly onto Dowex 1×8 resin, formate form [1 cm (outside diameter) × 10 cm, 100–200 mesh; experiments IIa-2, IIa-3, and IIb-1], or lyophilized and redissolved in 1.5 mL of water before being filtered onto the resin (experiments IIa-1 and IIb-2). Compounds were eluted with water (100 mL + 2 × 25 mL fractions) followed by 1.0 M formic acid (6 × 13 mL fractions). Fractions containing malic acid as judged by TLC were combined with the last preceding and first subsequent fractions and dried as described above for malate **I** to yield malate **II** (10–14 mg). The dry solid was dissolved in 0.5 mL of water, and two 100 μL aliquots were again withdrawn for radioactivity counting. Details for individual experiments are given in the Supporting Information Materials and Methods.

## Results

To investigate the stereochemical course of the Fom3 reaction at the transferred methyl group, N-terminally His_6_-SUMO-tagged Fom3 and N-terminally His_6_-tagged FomD from *Streptomyces wedmorensis* were expressed and purified from *E. coli*. As isolated, Fom3 contained 0.6 equiv of cobalamin. (*methyl*-*S*)- and (*methyl*-*R*)-l-(*methyl*-^2^H_1_)[*methyl*-^3^H_1_]methionine^54^**1a** and **1b** [hereafter termed (*methyl*-*S*)- and (*methyl*-*R*)-Met, respectively] were enzymatically converted on a relatively large scale (2 mM, 5 mL) using optimized conditions into the corresponding isotopologs **2a** and **2b**, respectively, of SAM, the methyl donor co-substrate in the Fom3 reaction (Figure 3). This reaction was catalyzed *in vitro* by N-terminally His_6_-tagged SAM synthetase (MetK) from *B. subtilis* obtained by heterologous expression in *E. coli*. Because the wild-type enzyme exhibits product inhibition, the previously described I317V variant^59^ was used to reduce the inhibition and maximize the yield of SAM from methionine.

Next, 30 μM Fom3, 0.25 mM hydroxocobalamin (HOCbl), 1 mM 2-HEP-CMP, and a reducing agent mixture (10 mM dithiothreitol, 4 mM NADH, and 1 mM methyl viologen) were added to the MetK reaction mixture to transfer the methyl group of SAM to 2-HEP-CMP, yielding (2*S*)-2-HPP-CMP **3a** and **3b** (Figure 3). Finally, FomD, the subsequent enzyme in the fosfomycin biosynthetic pathway,^19^ was used to hydrolyze (2*S*)-2-HPP-CMP to (*S*)-2-HPP (**4a** and **4b**) and CMP. The expected yield of **4a** and **4b** under these conditions was 5 μmol (0.7 mg) each, far less than the total mass of enzyme used in each three-step reaction sequence (13 mg, including 11 mg of Fom3).

To confirm full conversion of substrate to product under the reaction conditions described above, a replica reaction was also performed with l-(*methyl*-^13^C)methionine instead of chiral methyl-labeled methionine and the products were analyzed by ^31^P NMR spectroscopy (Figure S2). The only ^31^P signal visible in the phosphonate range (>5 ppm) was 2-hydroxy-(3-^13^C)propylphosphonate [(3-^13^C)-2-HPP], indicating full conversion of 2-HEP-CMP to (3-^13^C)-2-HPP by Fom3 and FomD. After confirming the success of the enzymatic reactions, we purified (*S*)-2-HPP products **4a** and **4b** by anion exchange chromatography (see Materials and Methods), lyophilized them, and converted them to acetates **5a** and **5b**, respectively, via Kuhn–Roth oxidation^63^ in a mixture of chromium trioxide and sulfuric acid in water, with modifications as described below.

Certain intermediates during the oxidation of 2-HPP to acetic acid, such as 2-oxopropylphosphonate, are susceptible to hydron exchange in the α-position in the acidic reaction medium. Exchange of an isotope at the chiral methyl group for H would lead to partially racemic (^1^H–^1^H exchange) or achiral species (^2^H– or ^3^H–^1^H exchange). To study the degree of isotope exchange, unlabeled racemic 2-HPP^61^ was oxidized in D_2_O with CrO_3_/H_2_SO_4_ under several different conditions. The degree of deuterium exchange was determined by NMR spectroscopy and/or high-resolution mass spectrometry of the acetates and their 4′-(phenyl)phenacyl derivatives (Scheme S1).^62^

Several reaction conditions were tested to slow deuterium exchange to acceptable levels. The originally published Kuhn–Roth reaction was refluxed, and the acetic acid was distilled at 1031 mbar.^63^ We decreased the reaction temperature to 50, 20, and 0 °C and performed the distillation at 1031 mbar at 50 and 20 °C and at ≤1 mbar (receiver flask was cooled with liquid nitrogen) for the reaction at 0 °C. The yield of mono- and dideuterated acetate formed could thus be decreased from 5.4% (CH_2_D) and 1.1% (CHD_2_) at 50 °C to ≤2% (combined) at 0 °C.

(*S*)-2-HPP **4a** derived from 10 μmol of (*methyl*-*S*)-Met **1a** was then subjected to the procedure described above to yield chiral, tritiated acetate **5a** [10 mg, containing 3.67 μmol of (2-^2^H_1_)[2-^3^H_1_]acetate and 7.33 μmol of unlabeled acetate in admixture with inorganic salts]. Due to the stoichiometric requirement of 2 equiv of SAM for each Fom3 turnover,^60^ only 5 μmol of 2-HPP and thus 5 μmol of labeled acetate were expected from each 10 μmol reaction mixture. Analogously, (*S*)-2-HPP **4b** derived from 10 μmol of (*methyl*-*R*)-Met **1b** gave acetate **5b** [9 mg, containing 4.13 μmol of (2-^2^H_1_)[2-^3^H_1_]acetate and 9.74 μmol of unlabeled acetate with inorganic salts]. The unlabeled acetate in **5a** and **5b** likely originated from impurities in **4a** and **4b**, respectively, after ion exchange chromatography; the resulting dilution of ^3^H activity was deemed acceptable, and subsequent procedures were otherwise unaffected.

**Figure 4:**
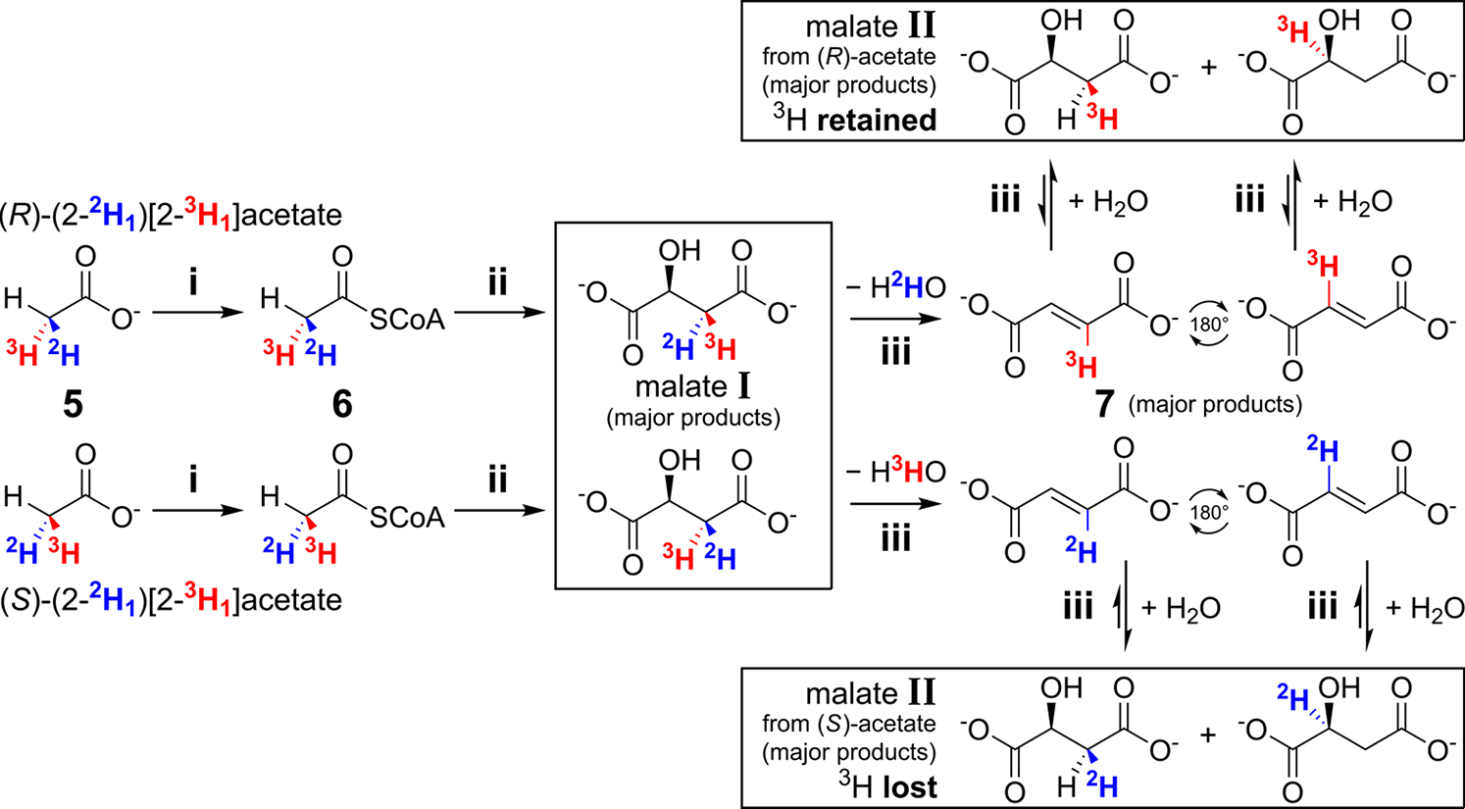
Enzymatic reactions for stereochemical analysis of chiral (2-^2^H_1_)[2-^3^H_1_]acetates **5a** and **5b**.^42,43^ Each initial sample of acetate was spiked with a small amount of [2-^14^C]acetate as an internal standard for determination of ^3^H in malates **I** and **II**. Malates **I** and **II** were isolated by anion exchange chromatography; intermediates acetyl-CoA **6** and fumarate **7** were generated and consumed *in situ*. Enzymes and co-substrates: (i) acetate kinase, ATP, coenzyme A; (ii) malate synthase, sodium glyoxylate; (iii) fumarase. Only the major products are shown for the malate synthase reaction (ii); ^1^H is preferentially removed by the enzyme due to a kinetic isotope effect (*k*_H_/*k*_D_ = 3.8).^65^ For a description of all ^3^H-containing products formed, see Figure S4. The ^3^H content of malate **II** after full equilibration indicates whether the process was started with (*S*)- or (*R*)-(2-^2^H_1_)[2-^3^H_1_]acetate (see Figure S4).

The acetates were stereochemically analyzed by the method of Cornforth and Arigoni, on the basis of reactions catalyzed by malate synthase and fumarase (Figure 4).^42,43^ In previous studies, the native malate synthase enzyme was purified from kilogram quantities of fresh commercial yeast or from a yeast strain genetically engineered to overproduce malate synthase.^41−43^ For this study, because the overproducing yeast strain is no longer available, malate synthase 1 from *S. cerevisiae* (ScMLS1) was expressed from a codon-optimized synthetic gene in *E. coli* and purified as an N-terminal His_6_ tag fusion. The tag was removed with thrombin, leaving three residues (GSH) at the N-terminus. Using modern molecular biology and affinity purification, 32 g of *E. coli* cells from 6 L of a standard LB culture yielded approximately 13000 units of malate synthase activity at 26 units/mg of protein, compared to the values of 5400 and 36 units/mg of protein obtained using more laborious methods in a typical preparation from 5 kg of fresh yeast.^64^

Recombinant ScMLS1 was incorporated into a chiral acetate analysis procedure adapted from ref (62). In short, 2 μmol of (2-^2^H_1_)[2-^3^H_1_]acetate **5a** [derived from (*methyl*-*S*)-Met **1a**] was spiked with [2-^14^C]acetate as an internal standard to a ^3^H/^14^C ratio of 3–4 and converted to (2-^2^H_1_)[2-^3^H_1_]- and [2-^14^C]malate **Ia** [experiment Ia-1 (Table 1)] in an enzyme system comprising acetate kinase, ATP, phosphotransacetylase, malate synthase, and sodium glyoxylate. Malate synthase preferentially abstracts a proton from the methyl group of the labeled acetyl-CoA (*k*_H_/*k*_D_ = 3.8) and adds the formal carbanion with inversion of configuration^42,43,65^ to the formyl group of glyoxylate to generate l-malate [termed malate **I** by Cornforth, Arigoni et al.;^42,43^ we use the same nomenclature here with specifiers **a** and **b** denoting the original (*methyl*-*S*)- and (*methyl*-*R*)-methionine isomers, respectively]. Figure 4 depicts the major components of malate **I** theoretically derived from pure (*R*)- and (*S*)-acetate **5**, produced by removal of ^1^H from the corresponding acetyl-CoA isomers **6**. The minor products resulting from removal of ^2^H (approximately 21% of each malate **I**) each contain ^3^H in the opposite position of the methylene group from the corresponding major products (Figure S4). Malate **Ia** with a ^3^H/^14^C ratio of 3.19 was isolated by anion exchange chromatography, crystallized, and counted. Then, malate **Ia** was equilibrated with fumarase to cause a *trans* elimination^66^ of H_2_O, H^2^HO, or H^3^HO depending on the stereochemistry at the methylene group of malate **Ia**, followed by reversible addition of H_2_O from the solvent to the resulting fumarate. The equilibrium mixture contains fumarate **7a** and malate **IIa**, which has undergone exchange of the C2 pro-*R* hydron with solvent and therefore has a ^3^H/^14^C ratio different from that of initial malate **Ia** (see Figure S4). Malate **IIa** was isolated in the same way as malate **Ia** and displayed a ^3^H/^14^C ratio of 0.79 (Table 1). The ^3^H/^14^C ratio of malate **IIa** divided by the ^3^H/^14^C ratio of malate **Ia** represents the fraction of ^3^H in malate **Ia** that was retained in malate **IIa**. This quantity is known as the *F* value^42,43,50^ and is diagnostic for whether acetate **5a** had the *R* configuration (*F* value near 80%) or the *S* configuration (*F* value near 20%) (see Figure S4). The experimentally observed *F* value was 24.8% for experiments Ia-1 (formation of malate **Ia**) and IIa-1 (equilibration with fumarase). This experimental sequence was repeated with twice the amounts of enzymes for the synthesis of malate **Ia**, which increased the radiochemical yield by 10% (experiment Ia-2). In a third experiment (Ia-3), acetate kinase and phosphotransacetylase were replaced by acetyl coenzyme-A synthetase. In both cases, the *F* values were similar to that from experiment IIa-1 (Table 1; mean *F* = 24.5% for experiments IIa-1, IIa-2, and IIa-3), demonstrating the robustness of the results with respect to the experimental conditions of acetate analysis.

**Table 1:** ^3^H/^14^C Ratios of Malates **I** and **II** Derived from Chiral (2-^2^H_1_)[2-^3^H_1_]Acetates **5a** and **5b** and Their Calculated *F* Values

	acetate **5a** derived from (*methyl*-*S*)-Met **1a**			acetate **5b** derived from (*methyl*-*R*)-Met **1b**	
experiment for malate **I**	Ia-1	Ia-2	Ia-3^a^	Ib-1	Ib-2
^3^H/^14^C in malate **I**	3.19	4.54	3.26	3.46	3.39
experiment for malate **II**	IIa-1	IIa-2	IIa-3	IIb-3	IIb-2
^3^H/^14^C in malate **II**	0.79	1.11	0.79	2.82	2.71
*F* value (%)	24.8	24.5	24.2	81.5	79.9
mean *F* value (%)	24.5			80.7	

a Acetate kinase and phosphotransacetylase were replaced by acetyl-CoA synthetase.

Similarly, a reaction mixture derived from (*methyl*-*R*)-Met **1b** was first converted to (2-^2^H_1_)[2-^3^H_1_]acetate **5b** and then to malates **Ib** and **IIb**. The *F* values of two parallel sets of experiments (Ib-1 followed by IIb-1 and Ib-2 followed by IIb-2) each performed with 3 μmol of **5b** spiked with [2-^14^C]acetate were 81.5% and 79.9% (mean *F* = 80.7%), respectively.

Mazacek^62^ and Arigoni reported an *F* of 21.4% for (*S*)-(2-^2^H_1_)[2-^3^H_1_]acetate and 82.4% for the *R* enantiomer for an enantiomeric excess (ee) of >96% for each compound, verified by ^3^H NMR spectroscopy. Similarly, Eggerer and Lenz^65^ used two different enzymatic methods to determine *F* values for pure (*S*)- and (*R*)-acetate of 21 ± 2% and 79 ± 2%, respectively. Therefore, acetate **5a** derived in this work from (*methyl*-*S*)-Met has an *S* configuration, and acetate **5b** derived from (*methyl*-*R*)-Met has an *R* configuration, which implies net retention of stereochemistry for the methyl transfer(s) from methionine to 2-HEP-CMP. As the formation of (2*S*)-2-HPP-CMP involves two methyl transfers, first from SAM to cob(I)alamin and then from MeCbl to 2-HEP-CMP, each step must occur with inversion, giving net retention. This result is concordant with findings from previous feeding experiments in which fosfomycin was extracted from *Streptomyces fradiae* supplemented with the same (*methyl*-*S*)- and (*methyl*-*R*)-Met.^41^ The small deviations of the *F* values from the values for enantiomerically pure acetates reflect an insignificant degree of racemization, which presumably occurs during Kuhn–Roth oxidation by exchange of the proton at the chiral methyl group for a proton from the reaction mixture.

## Discussion

Our chiral methyl analysis of the Fom3-catalyzed methyl transfer from SAM to 2-HEP-CMP demonstrates that this transformation occurs with net retention of stereochemistry at the methyl center under *in vitro* reconstituted reaction conditions. This finding is consistent with previous studies on *S. fradiae* cultures that were fed methionine with a stereodefined methyl group as a precursor to fosfomycin.^41^ These results, together with experiments showing that Fom3 catalyzes methyl transfer from SAM to B_12_,^17^ provide support for a mechanistic hypothesis in which MeCbl acts as an intermediate methyl carrier that is formed by S_N_2 displacement on SAM and that is consumed by methyl radical transfer during the Fom3 reaction cycle, each occurring with inversion of stereochemistry. Imfeld and Arigoni found that the chiral methyl groups of (^2^H_1_)[^3^H_1_]methylcobalamin in solution were more rapidly exchanged in the presence of a cob(I)alamin derivative than the latter reacted with an alkylating agent, causing complete racemization of the methyl group within minutes and thus preventing the synthesis of chiral (^2^H_1_)[^3^H_1_]methylcobalamin.^50,67^ When these findings are taken into account, during the Fom3 reaction cycle either cob(I)- or methylcob(III)alamin intermediates, or both, likely must remain enzyme-bound. Indeed, experiments in which isotopically differentiated MeCbl and SAM were both present in solution, and MeCbl was in excess, have shown that the methyl group installed by Fom3 on 2-HPP-CMP originates nearly completely from SAM.^17,18^ Thus, it appears that MeCbl in solution cannot compete with that formed on the enzyme and only the latter is used for productive turnover.

Our findings are also consistent with an earlier chiral methyl feeding study of *Streptomyces cattleya* that demonstrated retention of stereochemistry between methionine fed to the organism and the methyl group of the resulting thienamycin. Thienamycin biosynthesis also involves a B_12_-dependent rSAM enzyme,^25,48^ suggesting the Fom3 mechanism may be shared with other class B rSAM methyltransferases. This investigation represents, to the best of our knowledge, the first *in vitro* study of the stereochemical course of methyl transfer catalyzed by a member of this class of enzymes. The methods developed during this study may facilitate other such studies, including *in vitro* studies of family members that are believed to use S_N_2 chemistry instead of radical chemistry.^68−71^

## Supporting Information Available

Materials and Methods, sequences of codon-optimized genes, Figures S1–S4, and Table S1 (PDF)

## Accession Codes

Fom3, UniProtKB Q56184; FomD, UniProtKB O83033; MetK, UniProtKB P54419; ScMLS1, UniProtKB P30952; acetate kinase, UniProtKB P0A6A3; phosphotransacetylase, UniProtKB P39646; fumarase, UniProtKB P10173.

## Abbreviations

2-HEP-CMP: (5′-cytidylyl)-2-hydroxyethylphosphonate
2-HPP: 2-hydroxypropylphosphonate
2-HPP-CMP: (5′-cytidylyl)-2-hydroxypropylphosphonate
5′-dA: 5′-deoxyadenosine
5′-dA•: 5′-deoxyadenosyl radical
ATP: adenosine 5′-triphosphate
B_12_: cobalamin
CMP: cytidine 5′-monophosphate
CoA: coenzyme A
dr: diastereomeric ratio
HOCbl: hydroxocobalamin
IMAC: immobilized metal affinity chromatography
MeCbl: methylcobalamin
NADH: nicotinamide adenine dinucleotide (reduced)
NMR: nuclear magnetic resonance
RCY: radiochemical yield
rSAM: radical *S*-adenosyl-l-methionine
SAH: *S*-adenosyl-l-homocysteine
SAM: *S*-adenosyl-l-methionine
ScMLS1: *S. cerevisiae* malate synthase 1
SUMO: small ubiquitin-like modifier
TLC: thin-layer chromatography.
