# Supplementary material for: Overall Retention of Methyl Stereochemistry during B12-Dependent Radical SAM Methyl Transfer in Fosfomycin Biosynthesis
Source: Biochemistry. 2021 May 4;60(20):1587–96. doi: 10.1021/acs.biochem.1c00113 (PMC8158854; doi:10.1021/acs.biochem.1c00113)
Supplement: Supplementary file 1 — bi1c00113_si_001.pdf [file bi1c00113_si_001.pdf]

Supporting Information

Overall retention of methyl stereochemistry during B_{12}-dependent radical SAM methyl transfer in fosfomycin biosynthesis

Martin I. McLaughlin,[a] Katharina Pallitsch,[b] Gabriele Wallner,[c] Wilfred A. van der Donk,[d] and Friedrich Hammerschmidt*,[b]*

[a]*Department of Chemistry and Carl R. Woese Institute for Genomic Biology, University of Illinois at Urbana-Champaign, Urbana, Illinois 61801, United States*
[b]*Institute of Organic Chemistry, University of Vienna, 1090 Vienna, Austria*
[c] *Institute of Inorganic Chemistry, University of Vienna, 1090 Vienna, Austria*
[d]*Department of Chemistry and Carl R. Woese Institute for Genomic Biology and the Howard Hughes Medical Institute, University of Illinois at Urbana-Champaign, Urbana, Illinois 61801, United States*

Correspondence: vddonk@ilinois.edu ; friedrich.hammerschmidt@univie.ac.at

Contents

SI Materials and Methods S2

Sequences of codon-optimized genes S13

Figure S1. Plasmid map of btu-pBAD1030C-2. S15

Figure S2. ^{31}P NMR spectrum of a MetK/Fom3/FomD reaction performed with (*methyl*-^{13}C)methionine. S16

Figure S3. Glass apparatus used for Kuhn-Roth oxidation. S17

Figure S4. Major and minor products of enzymatic reactions during the configurational assay of chiral acetate. S18

Table S1. Radiochemical data for malates **I** and **II** isolated from chiral acetate samples. S19

References S20

SI Materials and Methods

Materials. Ethanolamine was obtained from Acros Organics via Thermo Fisher Scientific (Waltham, MA). Hydroxocobalamin hydrochloride (HOCbl), adenosine triphosphate disodium salt (ATP), chloramphenicol (Cam), 2-mercaptoethanol (βME), and glyoxylic acid monohydrate were obtained from Sigma-Aldrich (St. Louis, MO). Acetyl-coenzyme A lithium salt was purchased from CoALA Biosciences (Elgin, TX). Isopropyl β-D-thiogalactopyranoside (IPTG), ampicillin sodium salt (Amp), kanamycin sulfate salt (Kan), tris(2-carboxyethyl)phosphine hydrochloride (TCEP), tris(hydroxymethyl)aminomethane (Tris), and lysozyme were purchased from Gold Biotechnology (St. Louis, MO). Protease inhibitor E-64 and leupeptin hydrochloride were obtained from Cayman Chemical (Ann Arbor, MI). Phenylmethanesulfonyl fluoride (PMSF) was obtained from Fluka Chemical Corp (Ronkonkoma, NY), and DL-dithiothreitol (DTT) was from Promega (Madison, WI); *N*-(2-hydroxyethyl)piperazine-*N*′-ethanesulfonic acid (HEPES) was from Thermo Fisher Scientific (Waltham, MA). Tryptone was purchased from Dot Scientific (Burton, MI), yeast extract from IBI Scientific (Dubuque, IA), and sodium chloride (NaCl) from Santa Cruz Biotechnology (Dallas, TX). $CDCl_3$ (99.8%) and D_2O (99.9% D) were from Eurisotop (Cambridge, England) and Sigma-Aldrich (St. Louis, MO); 4′-(phenyl)phenacyl bromide, fumaric acid, CrO_3, $HClO_4$ (70%), formic acid (99%, 0.05% acetic acid) and indicator paper were from Merck (Darmstadt, Germany); L-malic acid (97%, ee 99%), concentrated H_2SO_4 (95-97%), sodium glyoxylate monohydrate, and Dowex 1X8 (Cl^- form, 100-200 mesh) were obtained from Aldrich or Sigma-Aldrich (Austria), and sodium formate from Fluka (Switzerland). TLC was carried out on 0.25 mm thick Millipore Sigma plates coated with silica gel 60 F_{254}. Spots were visualized by UV light and/or dipping the plate into a solution of $(NH_4)_6Mo_7O_{24}\cdot 4H_2O$ (25.0 g) and $Ce(SO_4)_2\cdot 4H_2O$ (1.0 g) in aqueous 10% H_2SO_4 (500 mL), followed by heating with a heat gun. Flash (column) chromatography was performed with Macherey-Nagel silica

gel 60 (230-400 mesh) (Düren, Germany). Cellulose-coated glass plates used during the isolation of malates by ion exchange chromatography (20 × 20 cm, without indicator F_{254}) were purchased from Merck. All other chemicals and solvents were of reagent grade or higher.

Expression of His_6-SUMO-Fom3. Expression and purification procedures were adapted from the previously published method.[1] coFom3-pSUMO was used to transform *E. coli* BL21(DE3) cells containing btu-pBAD1030C-2 and pDB1282. Overnight culture (4 × 40 mL) was inoculated into 4 × 4 L of M9-ethanolamine medium[2] + kanamycin (50 µg/mL) + ampicillin (100 µg/mL) + chloramphenicol (17µg/mL) + 1.5 µM HOCbl in which the trace metals mix was replaced by the metal mix of Studier.[3] Cultures were shaken at 37 °C and 180 rpm. At OD_{600} = 0.3, expression of Fe-S assembly and B_{12} uptake genes was induced by addition of 0.1% (w/v) solid arabinose, and the medium was supplemented with 25 µM $FeCl_3$ and 150 µM cysteine. At OD_{600} = 0.6, expression of His_6-SUMO-Fom3 was induced with 250 µM IPTG, and the medium was supplemented with an additional 25 µM $FeCl_3$ and 150 µM cysteine. After 18 h of expression at 18 °C and 180 rpm, 36 g of cells were harvested at 8,000 × *g*, flash frozen, and stored in liquid nitrogen.

Purification of His_6-SUMO-Fom3. Frozen cells (36 g) were resuspended in 75 mL of ice-cold lysis buffer (50 mM HEPES pH 7.5, 300 mM KCl, 20 mM imidazole, 5% (v/v) glycerol, 10 mM βME) supplemented with 1 mM PMSF, 1 mg/mL lysozyme, 100 U/mL DNase I (EMD Millipore), and 200 µM HOCbl. Cells were lysed while stirring on ice using a QSonica Q55 sonicator at 70% amplitude for 8 × 1 min; cell debris was removed by aerobic centrifugation in tubes sealed with electrical tape at 35,000 × *g* and 4 °C for 40 min. Clarified lysate was loaded onto a 1.5-cm diameter column containing 4.5 mL of HisPur Ni-NTA Superflow resin (Thermo Fisher), washed with 100 mL of ice-cold lysis buffer, and eluted with 10 mL of ice-cold elution buffer (lysis buffer with 300 mM imidazole and 20% (v/v) glycerol). Protein was concentrated aerobically in a sealed centrifugal filter device (EMD Millipore) to 2.5 mL,

exchanged into gel filtration buffer (50 mM HEPES pH 7.5, 300 mM KCl, 15% (v/v) glycerol, 5 mM DTT) using a PD-10 desalting column (GE Healthcare), and concentrated again to 750 µL. The concentrated protein was flash frozen and stored in liquid nitrogen.

Determination of His$_6$-SUMO-Fom3 cobalamin content. His$_6$-SUMO-Fom3 was diluted to 40 µM in 50 mM NaOH, mixed with an equal volume of 0.2 M KCN in 10 mM NaOH, and incubated at 95 °C for 30 min. The resulting dicyanocobalamin was quantified by UV spectrophotometry (ε_{367} = 30,800 M^{-1} cm^{-1})[4] in comparison to a standard curve of HOCbl treated in the same manner.

Expression and purification of *Bacillus subtilis* SAM synthetase (His$_6$-BsMetK I317V). Methods were adapted from Dippe *et al.*[5] coBsMetK[I317V]-pET28a was used to transform *E. coli* BL21(DE3) cells and 2 × 10 mL of starter culture was inoculated into 2 × 1 L of Luria-Bertani (LB) medium + kanamycin (50 µg/mL) shaken at 200 rpm and 37 °C. At an OD_{600} of 0.6-0.7, protein expression was induced with 50 µM IPTG and cultures were incubated at 20 °C for 14 h. Cells (10 g) were harvested at 8,000 × *g* and resuspended in 75 mL of lysis buffer (20 mM HEPES pH 8.0, 300 mM NaCl, 10 mM imidazole, 10% [v/v] glycerol) containing 0.3 mg/mL lysozyme, 1 mM PMSF, 1 µM protease inhibitor E-64, 1 µM leupeptin, and 1,000 U/mL DNase I. Cells were lysed by sonication for 8 × 1 min on ice and centrifuged at 38,000 × g and 4 °C for 80 min. Clarified lysate was loaded onto a 1.5-cm diameter column containing 2 mL of cobalt TALON resin (TaKaRa Bio USA) and washed with 100 mL of lysis buffer. Protein was eluted with 12 mL of elution buffer (20 mM HEPES pH 8.0, 300 mM NaCl, 250 mM imidazole, 10% [v/v] glycerol), concentrated to <2.5 mL, exchanged into oxygen-free storage buffer (100 mM HEPES neutralized with KOH, pH 8.0, 10% [v/v] glycerol) inside the anaerobic chamber using a PD-10 column, concentrated to <1 mL, flash frozen, and stored in liquid nitrogen.

Expression and purification of yeast malate synthase (ScMLS1). coScMLS1-pET28a was used to transform *E. coli* BL21(DE3) cells and 6 × 10 mL of starter culture was inoculated into 6 × 1 L of LB

medium + kanamycin (50 µg/mL). The cultures were shaken at 210 rpm and 37 °C; at an OD_{600} of 0.6, protein expression was induced with 50 µM IPTG and cultures were incubated at 20 °C and 210 rpm for 14 h. Cells (32 g) were harvested at 8,000 × *g* and resuspended in 200 mL of ice-cold lysis buffer (50 mM Tris pH 8.0, 20 mM KCl, 4 mM imidazole, 5 mM $MgCl_2$, 1 mM TCEP) containing 0.5 mg/mL lysozyme, 1 mM PMSF, and 100 U/mL DNase I. Cells were lysed by sonication for 8 × 1 min and the lysate was centrifuged at 38,000 × g and 4 °C for 75 min. Clarified lysate was loaded onto a 2.5-cm diameter column containing 16 mL of cobalt TALON resin equilibrated in lysis buffer. The column was washed with 100 mL of ice-cold lysis buffer and protein was eluted with 60 mL of ice-cold elution buffer (50 mM Tris pH 8.0, 20 mM KCl, 250 mM imidazole, 5 mM $MgCl_2$). Approx. 980 mg of His_6-ScMLS1 was concentrated to 30 mL and dialyzed against 4 L of thrombin cleavage buffer (50 mM Tris pH 8.0, 10 mM $CaCl_2$) at 4 °C overnight. The His_6-tag was then cleaved from the protein by adding 4,000 U of thrombin (Sigma-Aldrich). The thrombin cleavage reaction was incubated at 23 °C for 25 h. Thrombin was removed by running the reaction solution through 5 mL of *p*-aminobenzamidine agarose (Sigma-Aldrich) at a flow rate of 1 mL/min; 2 mM imidazole was added to the flow-through and the cleaved His_6-tag was removed by applying this solution to 16 mL of cobalt TALON resin equilibrated in thrombin cleavage buffer with 2 mM imidazole. The resin was washed with 20 mL of the same buffer, and the combined flow-through and wash fractions were concentrated to 30 mL. The resulting protein was divided into two 15-mL portions: the first portion was dialyzed against 1 L of malate synthase buffer (5 mM Tris-HCl pH 8.0, 10 mM $MgCl_2$) at 4 °C overnight, and the second portion was dialyzed in the same manner against 1 L of malate synthase buffer containing 15% (v/v) glycerol. The dialysis was repeated for both portions using fresh buffer for 4 h. Initial activity assays were performed with both portions; the enzyme in glycerol-free buffer contained 17.0 mg/mL protein with 490 U/mL of malate synthase activity (29 U/mg protein), and the enzyme in glycerol-containing buffer contained 19.9 mg/mL protein with 490 U/mL of malate synthase

activity (25 U/mg protein). The protein in glycerol-free buffer (approx. 220 mg in 14 mL) was supplemented with 34 mg/mL sucrose as a lyoprotectant, divided into 0.5-mL aliquots, frozen overnight at −20 °C, cooled to -80 °C for 1 h, cooled in liquid nitrogen, and then lyophilized. The protein in glycerol-containing buffer (approx. 300 mg in 15 mL) was flash frozen in 0.5-mL aliquots and stored at −80 °C. A single aliquot of each portion was then thawed, the lyophilized aliquot was reconstituted with 0.5 mL water, and both protein concentration and activity were measured for both aliquots. The lyophilized aliquot contained 15.1 mg/mL protein and 380 U/mL of malate synthase activity (25 U/mg protein), and the frozen aliquot contained 19.9 mg/mL protein and 540 U/mL of malate synthase activity (27 U/mg protein).

Optimization of the conversion of (±)-2-HPP to sodium acetate

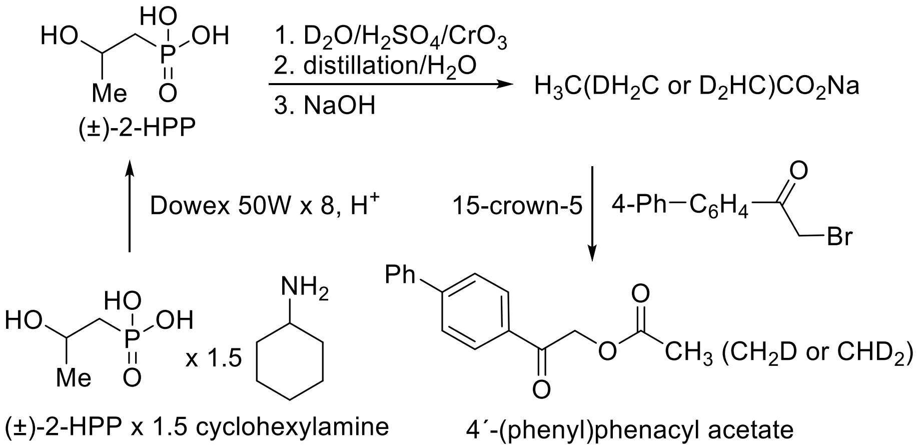

Scheme S1. Reaction sequence for the conversion of (±)-2-HPP to acetate and 4′-(phenyl)phenacyl acetate.

Preparation of 4′-(phenyl)phenacyl acetate from sodium acetate.[6] A mixture of powdered sodium acetate (30 mg, 0.366 mmol), 4′-(phenyl)phenacyl bromide (0.353 mg, 1.28 mmol, 3.5 equiv.), 15-crown-5 (50 mg, 0.227 mmol) and dry CH_3CN (5 mL) was heated at 80 °C. After stirring for 90 min, the reaction mixture was cooled and concentrated under reduced pressure. The residue was purified by flash-chroma-

tography [CH_2Cl_2/*n*-heptane = 3:1; R_f (CH_2Cl_2/*n*-heptane) = 2:1) = 0.21] to give 4′-(phenyl)phenacyl acetate (78 mg, 84%), which was crystallized from CH_2Cl_2/*n*-heptane to give colorless plates; m. p. 111 °C. ^{1}H NMR (600.25 MHz, $CDCl_3$): δ = 7.97 (d, J = 8.3 Hz, 2H), 7.69 (d, J = 8.3 Hz, 2H), 7.61 (d, J = 7.4 Hz, 2H), 7.46 (t, J = 7.4 Hz, 2H), 7.40 (t, J = 7.4 Hz, 1H), 5.25 (s, 2H), 2.23 (s, 3H, CH_3), phenacyl acetates derived from samples containing mono- and dideuterated acetate additionally displayed a satellite t at 2.217 (J = 2.2 Hz) for CH_2D and a satellite quint at 2.205 (J = 2.2 Hz) for CHD_2; ^{13}C NMR (150.93 MHz, $CDCl_3$): δ = 191.72, 170.42, 146.57, 139.58, 132.84. 128.97, 128.40, 128.32, 127.44, 127.24, 66.00, 20.57, 23.30 (CH_3), phenacyl acetates derived from samples containing mono- and dideuterated acetate additionally displayed a satellite t at 23.06 (J = 19.6 Hz) for CH_2D, and a satellite quint at 22.83 (J = 19.6 Hz) for CHD_2. HRMS-ESI (m/z, ±5 ppm): m/z calcd. for $C_{16}H_{14}NaO_3^+$: 277.084 (100.0%); found $[M+Na]^+$: 277.0832 (100%), $[M+1+Na]^+$: 278.0871 (19.4%), $[M+2+Na]^+$: 279.0918 (3.5%).

Conversion of L-(*methyl*-^{13}C)methionine to (2-^{13}C)acetate. Considering the low expected yield of acetate (0.3 mg) from each of the chiral methyl–labeled 2-HPP samples and the ubiquitous presence of acetic acid and acetate salts in common laboratory chemicals such as formic acid, a control sample of ^{13}C-labeled 2-HPP was oxidized to determine the yield of labeled acetate as well as any dilution with unlabeled acetate during the procedure. The (*S*)-(3-^{13}C)-2-HPP product of the MetK/Fom3/FomD replica reaction with L-(*methyl*-^{13}C)methionine was dissolved in water (3 mL) and (*S*)-(^{13}C)-2-HPP was isolated by anion exchange chromatography. The residue from the formic acid fraction was dissolved in deionized water (5 mL) and Kuhn-Roth oxidized under optimized conditions (using KOH for neutralization of acetic acid) to furnish potassium acetate (8 mg, slightly yellowish powder) which was analyzed by NMR spectroscopy. ^{1}H NMR (700.4 MHz, D_2O): δ = 1.93 ($^{12}CH_3$, integration: 1.00), satellite d (J = 127.1 Hz, $^{13}CH_3$, integration: 0.52); the sample thus contained potassium (2-^{13}C)acetate (3.7 µmol) and (2-^{12}C)acetate (6.6 µmol) in admixture with other impurities (mainly inorganic salts). The unlabeled acetate was very likely derived from remaining impurities in HPP after ion exchange chromatography.

Conversion of (*S*)-2-HPP 4a to potassium acetate 5a derived from (*methyl-S*)-L-(*methyl*-$^{2}H_1$)[*methyl*-$^{3}H_1$]methionine 1a. Tritiated (*S*)-2-HPP **4a** was isolated from the reaction mixture resulting from the enzymatic conversion of (*methyl-S*)-L-(*methyl*-$^{2}H_1$)[*methyl*-$^{3}H_1$]methionine **1a** using MetK/Fom3/FomD by anion exchange chromatography as described in Materials and Methods (reaction mixture A, 164,274 Bq of ^{3}H). The water fraction contained 80,984 Bq and the formic acid fraction (110 mg after lyophilization) 81,146 Bq of ^{3}H. The formic acid fraction was redissolved in deionized water (5.5 mL) and subjected to the optimized Kuhn-Roth oxidation using chromium trioxide (970 mg) and H_2SO_4 (0.56 mL, conc.). Neutralization and lyophilization produced potassium acetate **5a** [10 mg with ^{3}H activity of 72,909 Bq, equivalent to 3.67 µmol of acetate derived from tritiated methionine (radiochemical yield (RCY): 90%) in admixture with unknown impurities]. The combined amount of tritiated and unlabeled acetate was 11 µmol as determined by ^{1}H NMR spectroscopy using sodium *p*-toluene sulfinate as internal standard and a calibration curve for acetate concentrations between 0.24 µM and 2.4 µM. The lyophilized acetate was used to prepare a stock solution of **5a** (34.6 Bq/µL) by dissolving the salt (10 mg) in distilled water.

Conversion of 2-HPP 4b to potassium acetate 5b derived from (*methyl-R*)-L-(*methyl*-$^{2}H_1$)[*methyl*-$^{3}H_1$]methionine 1b. Analogously to (*S*)-2-HPP **4a**, (*S*)-2-HPP **4b** was isolated from reaction mixture B (168,706 Bq) by anion exchange chromatography. The water fraction contained 62,572 Bq and the formic acid fraction (oily residue, dissolved in 5.48 mL of deionized water for counting) 111,143 Bq of ^{3}H. The aqueous solution of **4b** was subjected to optimized Kuhn-Roth oxidation and furnished potassium acetate **5b** [9 mg, 74,222 Bq of ^{3}H, equivalent to 4.13 µmol of acetate derived from tritiated methionine (RCY: 67%) in admixture with unlabeled acetate (total acetate: 13.87 µmol; determined by ^{1}H NMR) and other unknown impurities]. A stock solution of **5b** (40.3 Bq/µL) was prepared by dissolving the salt (9 mg) in distilled water.

Conversion of potassium (2-$^2H_1$)[2-$^3H_1$]acetates 5 to malates I

Conversion of acetate 5a to malate Ia and isolation of malate Ia – experiment Ia-1. Potassium acetate **5a** (2 µmol, 352 µL of stock solution, 12,179 Bq of ^{3}H) was diluted in carbonate buffer and water and reagents were added as described in Materials and Methods. The resulting reaction mixture was spiked with sodium [2-^{14}C]acetate (44 µL of stock solution, equivalent to 3,344 Bq of ^{14}C) and malate synthase (10 U), phosphotransacetylase (18 U) and acetate kinase (7 U) were added. After 2 h of reaction, unlabeled malic acid and perchloric acid were added, the mixture was filtered onto Dowex 1×8 resin, and the resin was washed as described in Materials and Methods. The fractions eluted with 0.8 M formic acid (2 × 25 mL, numbered 1 and 2) and 1.0 M formic acid (6 × 25 mL, numbered 3 to 8) were each spotted 7 times on cellulose TLC plates. They were developed with Et_2O/HCO_2H (99%)/H_2O = 75:15:10 and dried in a vacuum desiccator at <1 mbar for 10 min. Then a solution of glucose (2 g) and aniline (2 mL) in a mixture of 1-butanol/ethanol/water 60:20:20 (100 mL) was applied. The plates were moved back and forth for 1 min to evaporate the liquid film from the surface. Finally, the plates were dried in a vacuum desiccator at <1 mbar for 15 min and heated in an oven at 140 °C for 10-15 min. Brown spots on a brownish background appeared; R_f = 0.60 for malic acid and 0.90 for fumaric acid; the latter stayed bound to the anion exchange resin. For stronger spots, small amounts (0.5 mL) of the aqueous fractions were put into vials (2 mL), cooled to 2-4 °C, then placed into a vacuum desiccator (15 mbar) over KOH and the water evaporated overnight. The residues were dissolved in water (50 µL, sonication for 30 s), and applied 5 times to a cellulose TLC plate as above. Malate **Ia** was detected in fractions 3, 4 and sometimes also 5 as judged by the intensity of the spots. Thus fractions 2 - 5 or sometimes even 2 - 6 were pooled, concentrated, and dried as described in Materials and Methods to yield crystalline malate **Ia** (23 mg); total activity calculated from ^{3}H: 7,440 Bq, from ^{14}C: 2,330 Bq, RCY: 70% (based on ^{14}C), ratio $^3H/^{14}C$ = 3.19 (experiment **Ia-1**, Table S1).

Experiment Ia-2. Experiment **Ia-1** was repeated except that the amount of [2-^{14}C]acetate was reduced (32 µL of stock solution, 2,432 Bq) and the amounts of all three enzymes were doubled (20 U malate synthase, 36 U phosphotransacetylase, 14 U acetate kinase) to determine whether the same results were obtained under these conditions. Activity of malate **Ia** (21 mg) from ^{3}H: 8,815 Bq, from ^{14}C: 1,940 Bq, RCY: 80% (based on ^{14}C), ratio ^{3}H/^{14}C = 4.54 **(**experiment **Ia-2**, Table S1).

Experiment Ia-3 – substitution of acetate kinase and phosphotransacetylase by acetyl-CoA synthetase. Experiment **Ia-1** was repeated except that the carbonate buffer (1 mL; 0.2 M sodium carbonate, pH 9.3, 8 mM $MgCl_2$, 2 mM K_3EDTA) was replaced by a phosphate buffer (1 mL; 100 mM KH_2PO_4, pH 7.4, 8 mM $MgCl_2$, 2 mM K_3EDTA) and acetate kinase and phosphotransacetylase were replaced by acetyl CoA synthetase (5 U). The pH of the mixture was adjusted to 7.46 with 0.2 M KOH before the addition of acetyl-CoA synthetase (5 U) and malate synthase (20 U). Activity of malate **Ia** (20 mg) from ^{3}H: 7,670 Bq, activity from ^{14}C: 2,350 Bq, RCY: 71% (based on ^{14}C), ratio ^{3}H/^{14}C = 3.26 (experiment **Ia-3**, Table S1).

Conversion of acetate 5b to malate Ib – experiment Ib-1. Potassium acetate **5b** (3 µmol, 297 µL of stock solution B, activity of ^{3}H: 11,969 Bq) and [2-^{14}C]acetate (39 µL, 2,964 Bq) were reacted in carbonate buffer (1 mL) with acetate kinase (21 U), phosphotransacetylase (54 U) and malate synthase (30 U) according to experiment **Ia-2**. Activity of malate **Ib** (26 mg) from ^{3}H: 8,450 Bq, from ^{14}C: 2,440 Bq, RCY: 82% (based on ^{14}C), ratio ^{3}H/^{14}C = 3.46 (experiment **Ib-1**, Table S1).

Experiment Ib-2. This experiment was a repetition of experiment **Ib-1**. Activity of malate **Ib** (22 mg) from ^{3}H: 8,430 Bq, activity from ^{14}C: 2,490 Bq, RCY: 84% (based on ^{14}C), ratio ^{3}H/^{14}C = 3.39 (experiment **Ib-2**, Table S1).

Conversion of malates I to malates II

Conversion of malate Ia derived from acetate 5a by experiment Ia-1 to malate IIa and its isolation - experiment IIa-1. Malate **Ia** from experiment **Ia-1** (dissolved in 0.8 mL of water, activity of ^{3}H: 5,952 Bq, activity of ^{14}C: 1,864 Bq) was equilibrated with fumarase, heated, lyophilized (^{3}H activity in collected water from lyophilization: 4,629 Bq), redissolved in 1.5 mL water, applied to Dowex 1×8 resin, and washed as described in Materials and Methods. Fractions eluted by the last 50 mL of water (2 × 25 mL, numbered 1 and 2) and 1.0 M formic acid (6 × 13 mL, numbered 3 to 8). Malate **IIa** was detected in fractions 5, 6 and 7 as judged by TLC. Fractions 4 – 8 were thus combined and yielded malate **IIa** (10 mg) [total activity calculated from ^{3}H: 1,189 Bq, from ^{14}C: 1,509 Bq, RCY: 81% (based on ^{14}C), ratio ^{3}H/^{14}C = 0.79] (experiment **IIa-1**, Table S1).

Experiments IIa-2 and IIa-3: Malates **Ia** from experiments **Ia-2** (dissolved in 0.8 mL, activity of ^{3}H: 7,052 Bq, activity of ^{14}C: 1,552 Bq, ratio ^{3}H/^{14}C = 4.54) and **Ia-3** (dissolved in 0.8 mL, activity of ^{3}H: 6,136 Bq, activity of ^{14}C: 1,880 Bq, ratio ^{3}H/^{14}C = 3.26 were converted to malates **IIa** analogously to experiment **IIa-1** except that the reaction mixtures were not lyophilized, but directly filtered onto the anion exchange resin. Experiment **IIa-2** furnished 12 mg of malate **IIa**: activity from ^{3}H: 1,233 Bq, from ^{14}C: 1,110 Bq, RCY: 72% (based on ^{14}C), ratio ^{3}H/^{14}C = 1.11. Experiment **IIa-3** furnished 12 mg of malate **IIa**: activity from ^{3}H: 1,285 Bq, from ^{14}C: 1,630 Bq, RCY: 87% (based on ^{14}C), ratio ^{3}H/^{14}C = 0.79 (experiments **IIa-2** and **IIa-3**, Table S1).

Conversion of malate Ib derived from acetate 5b by experiment Ib-1 to malate IIb – experiment IIb-1. Malate **Ib** from experiment **Ib-1** (dissolved in 0.8 mL, activity of ^{3}H: 6,760 Bq, activity of ^{14}C: 1,952 Bq, ratio ^{3}H/^{14}C = 3.46) was equilibrated with fumarase analogously to experiment **IIa-1** except

that the reaction mixture was not lyophilized, but directly filtered onto the anion exchange resin. Experiment **IIb-1** furnished 14 mg of malate **IIb**. Activity from ^{3}H: 4,340 Bq, from ^{14}C: 1,538 Bq, RCY: 79% (based on ^{14}C), ratio ^{3}H/^{14}C = 2.82 (experiment **IIb-1**, Table S1).

Experiment IIb-2: Malate **Ib** from experiment **Ib-2** (dissolved in 0.8 mL, activity of ^{3}H: 6,744 Bq, activity of ^{14}C: 1,992 Bq, ratio ^{3}H/^{14}C = 3.39) was equilibrated with fumarase analogously to experiment **IIa-1**. The reaction mixture was lyophilized and the tritiated water was collected (activity of ^{3}H: 1,498 Bq). Experiment **IIb-2** furnished 13 mg of malate **IIb**. Activity from ^{3}H: 3,505 Bq, from ^{14}C: 1,295 Bq, RCY: 65% (based on ^{14}C), ratio ^{3}H/^{14}C = 2.71 (experiment **IIb-2**, Table S1).

Sequences of codon-optimized genes:

coBsMetK[I317V] (SAM synthetase)

5'-
ATGAGCAAAAATCGTCGTCTGTTTACCAGCGAATCTGTGACCGAAGGCCATCCGGATAAAATTTGCGAT
CAGATTAGCGACAGCATTCTGGATGAAATCCTGAAGAAAGACCCTAACGCGCGTGTTGCTTGTGAAACT
AGCGTGACGACCGGTCTGGTTCTCGTGAGCGGTGAAATTACTACCAGCACGTATGTTGACATTCCGAAA
ACGGTTCGCCAGACCATTAAAGAAATCGGCTACACCCGTGCAAAATATGGTTTTGATGCGGAAACTTGC
GCGGTTCTGACGTCAATTGATGAGCAGAGCGCTGATATCGCGATGGGCGTTGACCAAGCGCTGGAAGCC
CGTGAAGGTACCATGAGCGATGAAGAAATTGAAGCAATTGGTGCGGGTGATCAGGGTCTGATGTTTGGT
TATGCGTGCAACGAAACGAAAGAGCTGATGCCTCTGCCGATTTCACTGGCCCATAAACTGGCCCGCCGT
CTGTCTGAAGTGCGCAAAGAGGATATTCTCCCGTACCTGCGTCCGGATGGCAAAACACAGGTGACGGTT
GAGTATGATGAAAATAACAAACCTGTGCGCATTGACGCGATTGTTATTTCAACTCAGCATCACCCGGAA
ATTACACTGGAACAAATTCAGCGCAACATTAAAGAACATGTAATCAACCCGGTTGTGCCGGAGGAACTG
ATTGATGAAGAAACTAAATATTTCATCAACCCTACGGGTCGTTTCGTGATCGGTGGCCCGCAAGGCGAT
GCGGGTCTGACTGGCCGCAAAATCATTGTCGATACCTACGGCGGTTATGCACGCCACGGCGGTGGCGCA
TTTAGCGGTAAGGACGCGACGAAGGTAGATCGTAGCGCAGCTTATGCGGCACGCTACGTTGCGAAAAAC
ATCGTGGCGGCTGAGCTGGCTGATAGCTGCGAAGTTCAGCTCGCATACGCGGTGGGTGTTGCACAGCCT
GTGTCTATTAGCATCAACACCTTCGGTTCTGGTAAAGCTAGCGAGGAAAAACTGATTGAAGTTGTGCGC
AATAACTTTGATCTCCGTCCTGCCGGCATTATCAAAATGCTGGATCTGCGCCGTCCGATCTATAAACAA
ACTGCTGCGTACGGCCATTTTGGTCGTCACGATGTTGACCTGCCATGGGAGCGCACCGACAAAGCGGAG
CAGCTGCGTAAAGAAGCGCTGGGTGAATAA-3'

coScMLS1 (malate synthase)

5'-
ATGGTGAAAGTGAGCCTGGATAACGTTAAACTGCTGGTGGACGTGGATAAAGAACCGTTCTTTAAGCCG
AGCAGCACCACTGTGGGTGACATTCTGACCAAAGATGCCCTGGAATTTATTGTTCTCCTGCATCGTACC
TTTAATAACAAACGCAAACAGCTGCTCGAAAATCGTCAAGTGGTTCAGAAGAAACTGGATAGCGGTAGC
TACCATCTGGACTTCCTGCCGGAGACGGCAAACATTCGTAATGATCCGACCTGGCAAGGTCCGATTCTG
GCACCTGGCCTCATCAATCGTAGCACGGAAATTACCGGCCCTCCGCTGCGTAACATGCTGATCAACGCC
CTGAATGCACCGGTTAATACCTACATGACGGATTTTGAAGACAGCGCCAGCCCGACCTGGAACAACATG
GTTTATGGTCAGGTGAACCTGTACGATGCGATTCGTAATCAGATTGACTTTGACACTCCTCGTAAGAGC
TACAAACTGAACGGTAATGTCGCAAATCTGCCGACCATTATCGTGCGCCCGCGTGGTTGGCACATGGTT
GAAAAACATCTGTATGTGGATGACGAACCGATCTCTGCCTCAATTTTCGATTTTGGCCTGTACTTTTAT
CACAATGCGAAAGAACTGATTAAACTGGGTAAAGGTCCGTACTTCTATCTGCCGAAAATGGAACATCAC
CTGGAAGCAAAACTGTGGAATGATGTGTTTTGCGTCGCGCAGGACTATATTGGCATTCCGCGCGGCACC
ATTCGCGCGACCGTCCTGATTGAAACCCTGCCGGCCGCATTTCAGATGGAAGAAATTATCTATCAGCTG
CGTCAGCACAGCTCAGGCCTGAATTGCGGTCGCTGGGATTATATTTTTAGCACGATTAAACGCCTGCGC
AACGACCCGAATCATATTCTGCCGAACCGTAACCAGGTGACGATGACCAGCCCGTTTATGGACGCGTAT
GTGAAGCGTCTGATTAACACCTGCCACCGTCGCGGTGTTCATGCCATGGGTGGCATGGCCGCGCAGATC
CCTATTAAAGATGATCCGGCCGCAAATGAAAAGGCGATGACTAAGGTGCGCAATGATAAAATTCGTGAA
CTGACCAATGGTCATGATGGTTCATGGGTTGCGCATCCGGCACTGGCGCCGATTTGCAACGAAGTTTTT
ATTAACATGGGCACCCCTAACCAGATTTACTTTATTCCGGAAAACGTTGTGACGGCCGCAAACCTCCTG
GAAACCAAAATTCCGAACGGCGAAATTACCACGGAAGGCATCGTGCAGAATCTGGATATTGGCCTGCAG

TATATGGAGGCGTGGCTGCGTGGTAGCGGCTGCGTTCCGATCAACAATCTGATGGAAGATGCAGCGACC
GCCGAAGTCAGCCGTTGCCAGCTGTATCAGTGGGTGAAACACGGCGTGACCCTGAAAGATACGGGTGAG
AAAGTTACCCCGGAACTGACTGAGAAAATCCTGAAAGAGCAGGTTGAACGCCTGTCAAAAGCGAGCCCT
CTGGGCGATAAGAACAAATTCGCACTGGCGGCCAAATACTTCCTGCCGGAAATTCGTGGTGAAAAGTTT
TCAGAGTTCCTGACGACCCTTCTGTATGATGAAATTGTGTCAACCAAAGCGACGCCGACCGATCTGAGC
AAACTGTAA-3'

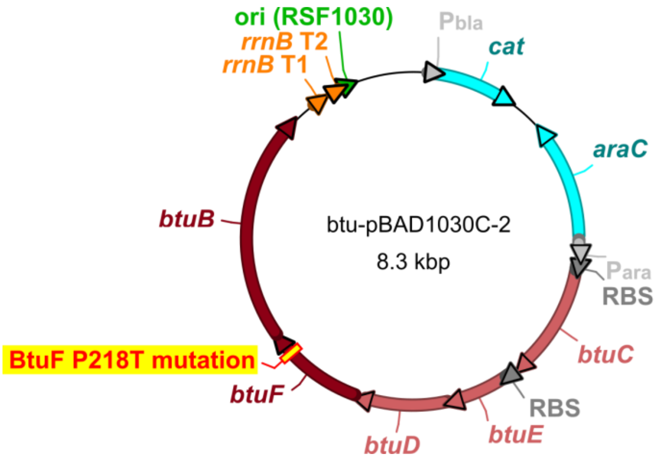

...CTATTAAGGAGGTAATTCATG-*btuC*-TAGCCG
CAAAAAGACGGTCTATGATTAAAAGCTAAGATTTTT
ACTGACCACTAAGGAGGTAAACGATG-*btuE*-TAAT
G-*btuD*-TGATG-*btuF*-TGATG-*btuB*-TGATCTA
GATTT...

Figure S1. Plasmid map of btu-pBAD1030C-2 encoding the *E. coli* B_{12} uptake genes *btuCEDFB*, used for coexpression with Fom3. Corrected from ref. 1 (see "Sequence of btu-pBAD1030C-2" in Materials and Methods).

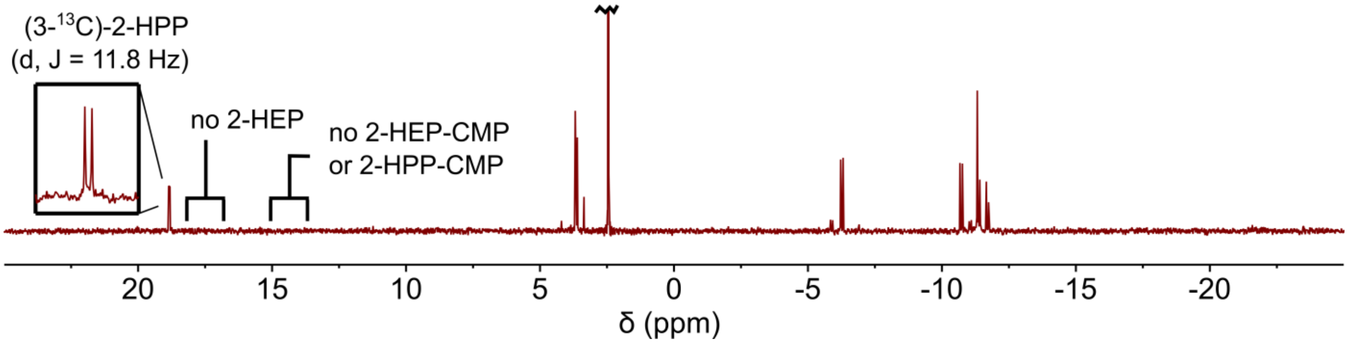

Figure S2. ^{1}H-decoupled ^{31}P NMR spectrum (242 MHz, D_2O) of the product mixture from a MetK/Fom3/FomD reaction performed with (*methyl*-^{13}C)methionine. The only visible signal in the phosphonate range (>5 ppm) of the ^{31}P NMR spectrum is that of (3-^{13}C)-2-HPP, which appears as a doublet due to coupling with ^{13}C at C3. Under these conditions, the signal for 2-HEP would appear at 17-18 ppm and the phosphonate signals for 2-HEP-CMP and 2-HPP-CMP would appear at 14-15 ppm.

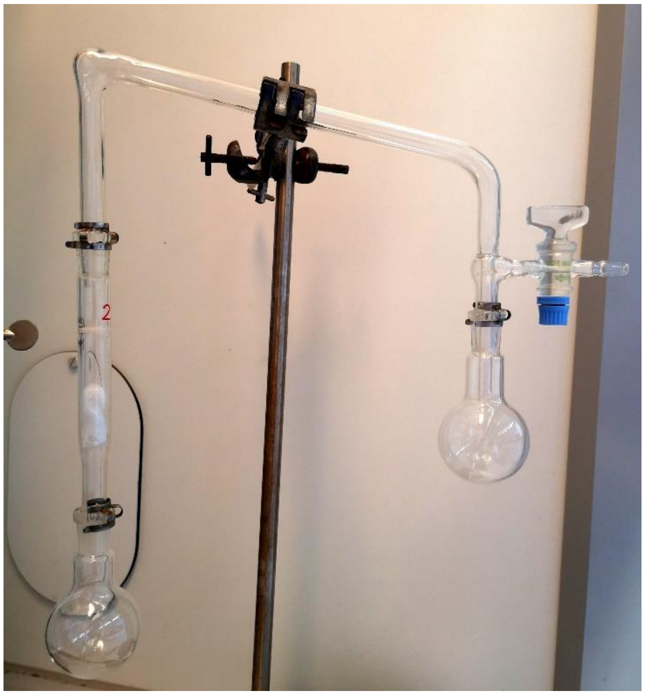

Figure S3. Glass apparatus (with two 50 mL round bottomed flasks, glass wool plug and frit of porosity 2) used for the performed Kuhn-Roth oxidation and subsequent vacuum distillation of acetic acid formed from 2-HPP.

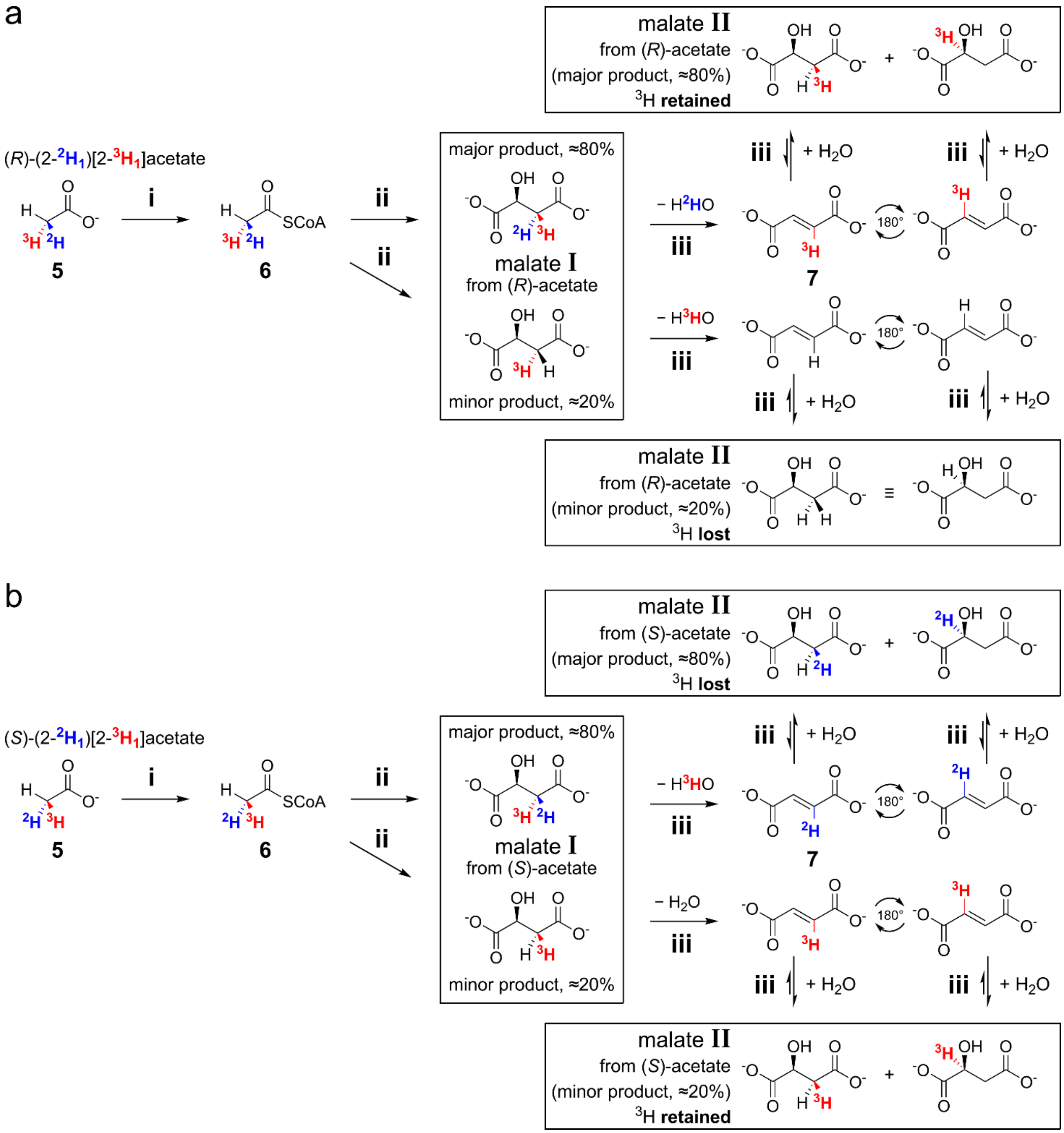

Figure S4. Major and minor products of enzymatic reactions during the configurational assay of (a) pure (*R*)-acetate and (b) pure (*S*)-acetate. The small amounts of malate **I** generated by the removal of ^{3}H from acetyl-CoA isomers **6** are not depicted; they do not contain ^{3}H and thus do not affect the assay. Compounds and reactions are labeled as in Figure 4. The finite intramolecular ^{2}H kinetic isotope effect on malate synthase (k_H/k_D = 3.8)[7] yields malate **I** as a mixture of species with opposite stereochemistry at C2. In turn, after full equilibration with fumarase, (*R*)-(2-$^2H_1$)[2-$^3H_1$]acetate will result in ~80% retention of the ^{3}H present in malate **I**, and (*S*)-(2-$^2H_1$)[2-$^3H_1$]acetate will result in ~20% retention of ^{3}H.

Table S1. Details of radiochemical data for malate **I** and **II**, F-values and mean F-values for chiral (2-$^{2}H_1$)[2-$^{3}H_1$]acetates **5a** and **5b**, derived from (*methyl-S*)- and (*methyl-R*)-(*methyl*-$^{2}H_1$)[*methyl*-$^{3}H_1$]methionine (**1a** and **1b**), respectively;

	(2-$^{2}H_1$)[2-$^{3}H_1$]Acetate **5a** derived from (*methyl-S*)-Met **1a**			(2-$^{2}H_1$)[2-$^{3}H_1$]Acetate **5b** derived from (*methyl-R*)-Met **1b**	
**Experiment for malate I**	**Ia-1**	**Ia-2**	**Ia-3**[a]	**Ib-1**	**Ib-2**
Activity from ^{3}H (Bq)	7,440	8,815	7,670	8,450	8,430
Activity from ^{14}C (Bq)	2,330	1,940	2,350	2,440	2,490
Radiochemical yield (RCY, %) of malate **I**[b]	70	80	71	82	84
$^{3}H/^{14}C$ in malate **I**	3.19	4.54	3.26	3.46	3.39
**Experiment for malate II**	**IIa-1**	**IIa-2**	**IIa-3**	**IIb-1**	**IIb-2**
Activity from ^{3}H (Bq)	1,189	1,233	1,285	4,340	3,505
Activity from ^{14}C (Bq)	1,509	1,110	1,630	1,538	1,295
Radiochemical yield (RCY, %) malate **II**[b]	81	72	87	79	65
$^{3}H/^{14}C$ in malate **II**	0.79	1.11	0.79	2.82	2.71
F-value (%)	24.8	24.5	24.2	81.5	79.9
**Mean F-value (%)**	**24.5**			**80.7**	

[a] acetate kinase and phosphotransacetylase were replaced by acetyl-CoA synthetase; [b] radiochemical yields (RCY) are based on ^{14}C.

References

[1] McLaughlin, M. I., and van der Donk, W. A. (2018) Stereospecific radical-mediated B_{12}-dependent methyl transfer by the fosfomycin biosynthesis enzyme Fom3, *Biochemistry 57*, 4967-4971.

[2] Bandarian, V., and Matthews, R. G. (2004) Measurement of energetics of conformational change in cobalamin-dependent methionine synthase, *Methods Enzymol. 380*, 152-169.

[3] Studier, F. W. (2005) Protein production by auto-induction in high-density shaking cultures, *Protein Expr. Purif. 41*, 207-234.

[4] Ljungdahl, L. G., LeGall, J., and Lee, J.-P. (1973) Isolation of a protein containing tightly bound 5-methoxybenzimidazolylcobamide (factor IIIm) from *Clostridium thermoaceticum*, *Biochemistry 12*, 1802-1808.

[5] Dippe, M., Brandt, W., Rost, H., Porzel, A., Schmidt, J., and Wessjohann, L. A. (2015) Rationally engineered variants of *S*-adenosylmethionine (SAM) synthase: reduced product inhibition and synthesis of artificial cofactor homologues, *Chem. Commun. 51*, 3637-3640.

[6] Mazacek, J. (1992) Dissertation ETH Nr. 9861, Zürich.

[7] Lenz, H., and Eggerer, H. (1976) Enzymic generation of chiral acetates. A quantitative evaluation of their configurational assay, *Eur. J. Biochem. 65*, 237-246.
